# News and Coming Events

**Published:** 1907-06-15

**Authors:** 


### NEWS AND COMING EVENTS.

The annual festival dinner of King's College Hospital will be held on Friday, June 28, at 7.30 p.m. in the Grand Hall at the Hôtel Cecil. Lord Methuen will take the chair.

Her Royal Highness Princess Louise, Duchess of Argyll, will lay the foundation-stone of the new East wing of the Church Army Central Headquarters, 53 Bryanston Street, Marble Arch, W., on Saturday, June 15, at three.

A festival banquet in aid of the Maintenance Fund of the Mount Vernon Hospital for Consumption and Diseases of the Chest will be held at the Hôtel Cecil on Monday, June 24, at 7.30 p.m. Mr. Henry Stedall will preside.

The Committee of University College Hospital issues a special appeal for increased support to the hospital. Since January 1, 1906, stock of the value of £12,286 4s. 6d. has had to be sold out, thereby seriously reducing the permanent income of the charity.

Dr. D. B. Lees, who has been conected with the staff of St. Mary's Hospital, Paddington, as physician, for the last twenty years, is retiring from the active staff and has been appointed Consulting Physician to the hospital. Dr. H. A. Caley succeeds him as full physician.

Dr. Frederick Hewitt, M.V.O., Emeritus Lecturer on Anæsthetics, will deliver a course of three lectures on "Certain Clinical Points in the Administration of General Anæsthetics," in the clinical theatre, London Hospital, on Fridays, June 14, 21, and 28, at 1 o'clock.

The Right Hon. Sir Wm. P. Treloar, Lord Mayor of London, supported by the Sheriffs, will preside at a festival dinner in aid of the funds of the Miller Hospital and Royal Kent Dispensary at De Keyser's Royal Hotel, Blackfriars, E.C., on Thursday, June 27, at 7.30. Tickets for the dinner (25s. each) can be obtained from the Secretary at the hospital.

The fourth annual "Ascot Ball" will be held in the Wharncliffe Rooms, Hôtel Great Central, on Wednesday, June 26. This year the Committtee has decided to devote the proceeds of the ball to the Maternity and District Nurses' Home, Plaistow. The secretary is Mr. G. D. White, Hôtel Great Central, N.W., to whom all communications should be addressed.

The *Kew Bulletin* announces that the Kew Museum has received dried specimens of *combretum sundaicum,* from a collector in Kuala Lumpur. The leaves of this plant are chewed by Chinamen as an anti-opium remedy, and are said to contain an active principle which counteracts the action of that drug. No analysis of the specimens has as yet been made.

The annual general meeting of the Hospital and Home for Incurable Children was held on Thursday, May 30, Canon Duckworth presiding. The report showed a debt of £6,307, against which stands as asset the ground rent of £250 a year. With the rental of the stabling now let on lease the committee hopes to secure a fixed income of £405 a year, which, when once secured, will make the position of the hospital so sound that the "Out-patients' Fund" can be taken up and continued, and an endeavour made to provide hospital and home accommodation for the children after they leave the institution at the age of 16, for as long as they want help, or for as long a time as is thought desirable. During the year the average cost per child was 18s. 8d. a week, as against the 18s. 6½d. of 1905.

The following is a list of lectures, which are free to medical practitioners, to be delivered during June and July on Thursdays, at 4 p.m., at the Hospital for Sick Children, Great Ormond Street, W.C.: June 20, Mr. Kellock on "Affections at and about the Umbilicus"; June 27, Dr. Garrod on "Diabetes in Children"; July 4, Dr. Still on "Bilious Attacks in Children"; July 11, Dr. Thursfield on "The Diagnosis, Prognosis, and Treatment of Pleural Effusions in Children"; and July 18, Mr. Lane on "Fractures."

We are glad to welcome the first number of *The Journal of Clinical Research,* the official organ of the Clinical Research Association, Limited, Watergate House, Adelphi, W.C. The valuable work that the Association is doing demands some recognition, and this official organ should bring it prominently before the profession. The *Journal,* we note from the introductory article, "is designed to provide for the solitary worker in private practice a substitute for the companionship of colleagues and students which keeps the hospital physician and surgeons abreast of all advances in laboratory aids to diagnosis." The first number is interesting and gives many practical hi[illegible] which will be most useful to the general practitioner.

The annual election of the Council of the Royal College of Surgeons of England will be held on Thursday, July [illegible] next. The retiring members of the Council are Sir John Tweedy, Mr. Page, Mr. Mansell Moullin, and Mr. Frederic Eve, the two last being "substitute members of Council." Sir John Tweedy does not intend to offer himself for re-election, but four new candidates are in the field—namely, Mr. Charles Higgens (Consulting Ophthalmic Surgeon, Guy's Hospital), Mr. Bruce Clark (Surgeon to St. Bartholomew's Hospital), Mr. Charters Symonds (Surgeon to Guy's Hospital), and Mr. W. Spanton (Consulting Surgeon to the North Staff. Infirmary. The poll will be open at the College between the hours of three and five for personal voting.

